# Methylglyoxal: Antimicrobial activity against blood culture isolates of Salmonella Typhi and other Gram negative rods

**DOI:** 10.12669/pjms.35.4.807

**Published:** 2019

**Authors:** Raja Kamran Afzal, Fizza Khalid, Abdul Hannan, Syed Azhar Ahmed

**Affiliations:** 1 Dr. Raja Kamran Afzal, FCPS, PhD. Department of Pathology, Armed Forces Institute of Cardiology, Rawalpindi, Pakistan; 2 Fizza Khalid, Department of Pathology, University of Health Sciences, Lahore, Pakistan; 3 Dr. Abdul Hannan, Dip Card, FRCPath. Department of Pathology, University of Health Sciences, Lahore, Pakistan; 4 Dr. Syed Azhar Ahmed, FRCPath, PhD. Department of Pathology, Baqai Medical University, Karachi, Pakistan

**Keywords:** Blood culture, Gram negative rods, Methylglyoxal, Salmonella Typhi

## Abstract

**Objectives:**

To evaluate the activity of Methylglyoxal against the blood culture isolates of Salmonella Typhi and various Gram negative rods and to compare the activity of Methylglyoxal against S. Typhi and other Gram negative rods.

**Methods:**

It was an experimental study conducted at the Department of Microbiology, University of Health Sciences (UHS), Lahore-Pakistan in collaboration with the Department of Microbiology, CMH Lahore, from July 2011 to June 2012. Recent blood culture isolates of *S. Typhi* and other Gram negative rods were collected from different hospitals of Lahore and kept stored at -80°C. As per the latest CLSI guidelines, morphological, biochemical and serological identification was carried out and antimicrobial susceptibility was tested. A multi-point inoculator was used to carry out agar dilution for determination of MICs of MGO. Results were determined after compilation of data using latest SPSS version.

**Results:**

MIC_90_ of MGO against the clinical isolates of S. Typhi was 0.20 *mg/mL* (2.8 *mM*) and against Gram negative rods it was 0.21 *mg/mL* (3.0 *mM*). The p-value of MICs of MGO against the isolates of S. Typhi was 0.023 when compared with Gram negative rods (p<0.05; statistically significant).

**Conclusion:**

MGO has a scientifically proven *in vitro* antimicrobial activity against blood culture isolates of S. Typhi and various Gram negative rods.

## INTRODUCTION

Medical research is continuing towards administration of honey for systemic infections, particularly where conventional therapy is failing. On the basis of its prominent antibacterial activity, honey is being chosen by physicians naturally as an alternate antimicrobial compound.^1^ The ‘bee-origin factors of honey are its increased osmolarity, reduced pH and H_2_O_2_ released by glucose oxidase. Second is methylglyoxal (MGO), which is the non-peroxide factor of ‘plant-origin’. These are its main contributing antibacterial factors.^2^ With the help of chemiluminescence-HPLC, a portion of New Zealand manuka nectar has been isolated which shows antimicrobial activity in a non-peroxide way.^3^ As compared to the other flora of honeys, there is atleast 100-folds higher concentration of MGO in manuka honey (*Leptospermum scoparium*). By modifying the structure of target molecules, MGO plays a role in the pathogenesis of impaired diabetic wound healing.^4^ The conversion kinetics of dihydroxyacetone (DHA) to MGO were studied in mānuka and other honeys and it was found that MGO originates from a change in an abundantly present compound called DHA in the nectar of manuka flowers.^5^ The strength and impact of an antimicrobial agent in honey is dependent upon the concentration of MGO in it. Previously the power of manuka honey has been rated by the nectar producers as Unique Manuka Factor (UMF). Because of its potent antimicrobial properties, manuka honey having higher UMF is in high demand.^6^ Advanced glycation end products (AGEs) of protein and DNA give rise to a highly reactive precursor and a dicarbonyl compound called MGO. Newer experiences demonstrate that higher levels of MGO, the MGO derived AGEs (MGO-AGE) and hydroimidazolone-1 (MGO-H1), along with dysfunctioning framework of glyoxalase are connected to a few age-related medical issues including certain cardiovascular problems, diabetes, disorders of central nervous system and cancer. MGO has been demonstrated to act by developing AGEs and glutathione adducts in cytoplasm of the cells, including microscopic organisms.^7^ Recently, some antibiotics have also shown synergistic effects when used in the presence of MGO, e.g., with linezolid against *S. aureus*.^8^ It has also been proved lately that MGO is a potent *in vitro* antimicrobial agent against both planktonic and biofilm producing bacteria like *S. aureus* and *P. aeruginosa*, while being safe to the mucosal surface.^9^ The vast potential for usage and application of MGO as the non-preoxide part of medical grade honey within the medical field still remains unexplored.

Typhoid is still a major health issue causing mortality and morbidity in developing countries. About 11 to 21 million new patients globally and between 128,000 and 161,000 deaths due to typhoid occur per year, as stated by World Health Organization lately. About 93% of these cases are reported from Asia.^10^ Reduced ciprofloxacin susceptibility and the emergence of multidrug resistance in S. Typhi have rendered conventionally used anti-typhoid drugs like ampicillin, chloramphenicol and co-trimoxazole ineffective for treatment. The real prospect to be faced is that untreatable typhoid fever is likely to emerge sooner or later.^11^ After fluoroquinolone and multi-drug resistance, the rapidly spreading ceftriaxone resistance allover Pakistan is a big cause of concern for the public health and medical authorities, which has been classified as extensively drug resistant (XDR Typhoid).^12^ Consequently, efforts have to be made to evaluate new agents having potent antibacterial activity to be used in patients having MDR and XDR Typhoid.

Gram-negative organisms cause bloodstream infections, pneumonia, meningitis, and surgical site infections (SSI) in the healthcare setup. These organisms have the ability to transfer genetic material that can make other bacteria resistant to antimicrobials. They also find novel ways to be resistant to antimicrobials due to their built-in abilities. In most of the infections caused by Gram-negative organisms, the therapeutic options have become limited to a critical level. Most of the new antimicrobial agents are in the planning stage, and a considerably good potential remains unexplored in this regard.^13^ This study aims at evaluating the *in vitro* antibacterial activity of MGO against the blood culture isolates of S. Typhi and various other Gram negative rods.

## METHODS

It was an experimental study conducted at the Department of Microbiology, University of Health Sciences (UHS), Lahore-Pakistan in collaboration with the Department of Microbiology, CMH Lahore, from July 2011 to June 2012. Microbiological procedures were carried as per recent CLSI guidelines having relevant positive and negative controls. Recently isolated 157 *S. Typhi* and 33 Gram negative rods from specimens of blood culture were collected from various hospitals of Lahore and were stored at -80°C. After thawing and sub-culturing on recommended culture media, Gram stain was done and colony morphology was observed to identify the isolates on preliminary basis. Catalase and oxidase enzymes production were tested and motility was observed by hanging drop method. API-20 E (bioMerieux, France) panels were used to perform biochemical identification. O, H and Vi (BD Difco, USA) polyvalent and group specific Salmonella antisera were used to identify them serologically. Disc diffusion method (Kirby-Bauer) was used to carry out antimicrobial susceptibility testing of the isolates on Meuller-Hinton agar (MHA) (Oxoid, UK). Ampicillin (10µg), co-trimoxazole (1.25/23.75µg), chloramphenicol (30µg), nalidixic acid (30µg), ciprofloxacin (5µg), ceftriaxone (30µg), aztreonam (15µg) and imipenem (10µg), antibiotic discs (Oxoid, Basingstoke, UK) were applied with their respective disc contents and zones of inhibition were noted in millimeters (mm) after incubation at 35°C aerobically for 18-24 hours. MP Pharma (29525 Fountain Parkway Solon, OH 44139, USA) was the authorized supplier from where MGO was purchased. Its expiry and sterility were confirmed before use. MGO was tested for its MICs by agar dilution method against 157 blood culture isolates of S. Typhi, 33 Gram negative rods and 6 ATCC (American Type Culture Collection) control stains. For inoculating multiple isolates simultaneously, multi-point inoculator (Mast Diagnostics, England) was used. For incorporation into the MHA, serial dilutions of MGO were made from 0.08 *mg/mL* to 0.24 *mg/mL*, based on its molecular weight 76.02 and these plates were incubated aerobically at 35°C for 18-24 hours. ATCC control strains were used to monitor the study as positive control strains and indigenous Gram negative organisms were also used to compare and validate this experimental work.^14^

The tested GNR isolates (*n*=33) include *Escherichia coli* (*n*=18), *Klebsiella pneumoniae* (*n*=6), *Enterobacter cloacae* (*n*=2), *Klebsiella oxytoca* (*n*=2), *Citrobacter braakii* (*n*=2), *Enterobacter agglomerans* (*n*=2) and *Enterobacter aerogenes* (*n*=1). The ATCC control strains (*n*=6) used were *Staphylococcus aureus* ATCC-25923 (*n*=1), *Escherichia coli* ATCC-25922 (*n*=1), *Klebsiella pneumonia*e ATCC-700721 (*n*=1), *Salmonella typhimurium* ATCC-39183 (*n*=1), *Acinetobacter baumanii* ATCC-19606 (*n*=1) and *Klebsiella oxytoca* ATCC-700324 (*n*=1). For internal quality control three MHA plates were used. First one contained MHA with MGO incorporated in it, second plate had MHA with incorporated MGO but inoculated with sterile pins of the multi-inoculator, and the third plate contained MHA without MGO but inoculated by pins of the multi-inoculator impregnated with the test and control isolates. When observed with an unaided eye and against a dark background, the MIC was determined as the least concentration of MGO at which there was no bacterial growth visible on surface of the agar.

The data was interpreted according to defined criteria of MIC range, MIC_50_ and MIC_90_. The data was further sorted out by SPSS v.22. Independent sample t-test was applied to see the difference in antibacterial activity of MGO among all groups of isolates independently. The results were considered to be statistically significant at p-value <0.05.

## RESULTS

The first two internal control plates did not show any growth but the third one showed growth of all organisms. All ATCC control strains also showed growth. A total of 157 S. Typhi and 33 Gram negative rods isolates were tested against a panel of antibiotics. The range of MICs of MGO against S. Typhi group was 0.14 to 0.24 *mg/mL* (2.0 to 3.4 *mM*), while it was slightly narrower 0.16 to 0.22 *mg/mL* (2.3 to 3.1 *mM*) against Gram negative rods group. The MIC_90_ of MGO against S. Typhi group was 0.20 *mg/mL* (2.8 *mM*), while it was slightly higher against Gram negative rods group 0.21 *mg/mL* (3.0 *mM*) (Tables-I and II).The Mean ± SD of S. Typhi group was 0.05 *mg/mL* (0.7 *mM*), whereas, the Mean ± SD of GNR group was 0.03 *mg/mL* (0.4 *mM*). Independent sample t-test showed that the p-value of MICs of MGO against S. Typhi group when compared to other Gram negative rods group was 0.023 (p<0.05; statistically significant), with Mean ± SD 0.04 ± 0.02.

**Table I:** MICs of MGO against S. Typhi (n=157).

	MICs of MGO			Mean ± SD

	Range	MIC_50_	MIC_90_	
In mg/mL	0.14 to 0.24	0.19	0.20	0.05
In mM	2.0 to 3.4	2.7	2.8	0.7

**Table II:** MICs of MGO against other GNR Isolates (n=33).

	MICs of MGO			Mean ± SD

	Range	MIC_50_	MIC_90_	
In mg/mL	0.16 to 0.22	0.20	0.21	0.03
In mMx	2.3 to 3.1	2.8	3.0	0.4

## DISCUSSION

Based on MIC and MBC, it has been demonstrated that honey and its components show a superb antimicrobial action against *S. aureus, E. faecalis, P. aeruginosa, E. coli*, and many other clinical pathogens including fungal isolates in combating infections due to these microorganisms in hospital practice.^15^ Increased virulence in S. Typhi may be due to the presence of other virulence genes on R-plasmid. In Pakistan, high mortality and severe complicated illnesses have been reported due to circulating strains of MDR and XDR S. Typhi.^16^

In this study, the MIC_90_ of MGO against S. Typhi isolates was 0.20 *mg/mL* (2.8 *mM)*, and GNR isolates having *E. coli* strains (*n*=18) was 0.21 *mg/mL* (3.0 *mM*) and control strains of *E. coli* ATCC 25922 was 0.20 *mg/mL* (2.8 *mM*) and *S. aureus* ATCC 25923 was 0.15 *mg/mL* (2.2 *mM*). These values are relatively but proportionately high when compared to a study on MGO, where *E*.*coli* and *S. aureus* had comparatively lower MIC of 0.08 *mg/mL* (1.1 *mM*), as analyzed using the agar well diffusion assay. The studied samples of manuka honey showed antibacterial activity in MGO concentrations of 1.1 to 1.8 *mM* which corresponded when it was diluted to 15 to 30%, whereas most of the other investigated honeys showed no antibacterial effect in dilutions of up to 80% (v/v with water) or below. This study also validated that MGO is unambiguously the predominant non-peroxide antibacterial agent in manuka honey.^17^ Method used for MIC determination in this study was agar dilution, which likely caused the comparative difference. Agar well diffusion method for determination of MIC used by Mavric *et al*. lacks sensitivity and proper standardization as compared to the agar dilution method, thus affecting the outcome of assay.^17^,^18^ MGO, a phytochemical compound was inducted in a hydrogel for its topical antibacterial action. Their MICs against *S. aureus* and methicillin-resistant *S. epidermis* (MRSE) were found comparatively lower 0.07 *mg/mL* (1.05 *mM*). Thus a stable and active combination was prepared which was appropriate for the prophylaxis and treatment of surgical wounds and burns. The MIC_90_ values 0.20 *mg/mL* (2.8 *mM*) in this study were comparable with a variation towards higher side, but broth dilution assay had been used for determination of MIC. Moreover, in this study the isolates belonged to different genera. MGO showed comparable MIC of 0.15 *mg/mL* (2.2 *mM*) against *S. aureus* ATCC 25923 which was used as a control isolate in this study.^19^ Taking a lead, many researchers impregnated the substrate of non-woven wound dressings with manuka honey or MGO. To facilitate a good antimicrobial effect against *S. aureus* and *K. pneumoniae*, the range of MGO concentration required was 0.0170 to 0.1 mg/cm^2^. Against the three prevalent wound and nosocomial pathogens, i.e., *S. aureus*, *P. aeruginosa* and *E. faecalis*, the liquid form MIC and MBC were also evaluated for MGO.

In order to achieve a good bacteriostatic or bactericidal effect in materials other than the MGO-impregnated fabrics, the solutions with comparatively much higher MGO concentrations in the range of 128 *mg/L* to 1024 *mg/L* were required. The importance of the MGO-based impregnation as environmental friendly and antibiotic-free wound dressings was proved scientifically by comparison.^20^

The range of MIC of MGO against S. Typhi in this study was 0.16 to 0.22 *mg/mL* (2.3 to 3.1 *mM*), it is however comparable to an effective concentration (EC) range of MGO against a Gram negative rod *P. aeruginosa* was 0.15 to 1.2 *mg/mL* (2.1 to 16.4 *mM*), and against *S. aureus* was 0.08 to 0.3 *mg/mL* (1.1 to 4.3 *mM*). EC against sessile (biofilm-forming) *P. aeruginosa* ranged from 0.5 to 3.6 *mg/mL*. *S. aureus* and *P. aeruginosa* are the known organisms that cause formation of polysaccharide biofilms on implanted devices or tissues in post-surgical patients. Biofilms render the antibiotics impenetrable, thus leading to therapeutic failure. MGO is a potent antibacterial agent against both the planktonic and sessile (biofilm-forming) organisms.^21^ Under non-physiologic conditions, the results of some of the MGO in-vitro experiments have been extrapolated to the in-vivo conditions, but with varied and confusing conclusions. However, the overall potential beneficial effects of MGO clearly outweigh its possible toxic role in *in vivo* conditions, meriting it to be utilized cautiously for the benefit of ailing humanity at this stage.^22^ However, the potential topical and systemic safety profile of MGO needs to be established in order for it to be used as a prospective antibacterial agent.

## CONCLUSION

MGO has been observed to have a scientifically proven *in vitro* antibacterial activity against blood culture isolates of S. Typhi and various genera of Gram negative rods.

### Authors’ Contribution

**RKA:** conception, literature searchstudy design, questionnaire design, acquisition of data, data collection, study performance, gathering of results, their interpretation and analysis. Drafting, editing, finalizing, agreed to be accountable for all aspects of the work.

**FK:** study design, data collection, performance, results, their analysis and interpretation, drafting and finalizing, agreed to be accountable.

**AH and SAA:** Conception, literature search, study design, interpretation of data, final approval of draft, agreed to be accountable.
